# Adding simultaneous integrated boost to whole brain radiation therapy improved intracranial tumour control and minimize radiation-induced brain injury risk for the treatment of brain metastases

**DOI:** 10.1186/s12885-023-11739-9

**Published:** 2023-12-16

**Authors:** Kunning Zhang, Tian Zhang, Zhoubo Guo, Fangdong Zhao, Jiacheng Li, Yanqi Li, Yang Li, Xiaoyue Wu, Xi Chen, Wencheng Zhang, Qingsong Pang, Ping Wang

**Affiliations:** Departments of Radiation Oncology, Tianjin Medical University Cancer Institute & Hospital, National Clinical Research Center for Cancer, Tianjin’s Clinical Research Center for Cancer, Key Laboratory of Cancer Prevention and Therapy, Huanhu West Road, Hexi District, Tianjin, China

**Keywords:** Brain metastasis, Whole-brain radiation therapy, Simultaneous integrated boost, Local tumour control, Radiation-induced brain injury

## Abstract

**Background:**

Brain metastases (BMs) are the most frequent intracranial tumours associated with poor clinical outcomes. Radiotherapy is essential in the treatment of these tumours, although the optimal radiation strategy remains controversial. The present study aimed to assess whether whole brain radiation therapy with a simultaneous integrated boost (WBRT + SIB) provides any therapeutic benefit over WBRT alone.

**Methods:**

We included and retrospectively analysed 82 patients who received WBRT + SIB and 83 who received WBRT alone between January 2012 and June 2021. Intracranial progression-free survival (PFS), local tumour control (LTC), overall survival (OS), and toxicity were compared between the groups.

**Results:**

Compared to WBRT alone, WBRT + SIB improved intracranial LTC and PFS, especially in the lung cancer subgroup. Patients with high graded prognostic assessment score or well-controlled extracranial disease receiving WBRT + SIB had improved intracranial PFS and LTC. Moreover, WBRT + SIB also improved the long-term intracranial tumour control of small cell lung cancer patients. When evaluating toxicity, we found that WBRT + SIB might slightly increase the risk of radiation-induced brain injury, and that the risk increased with increasing dosage. However, low-dose WBRT + SIB had a tolerable radiation-induced brain injury risk, which was lower than that in the high-dose group, while it was comparable to that in the WBRT group.

**Conclusions:**

WBRT + SIB can be an efficient therapeutic option for patients with BMs, and is associated with improved intracranial LTC and PFS. Furthermore, low-dose WBRT + SIB (biologically effective dose [BED] ≤ 56 Gy) was recommended, based on the acceptable risk of radiation-induced brain injury and satisfactory tumour control.

**Trial registration:**

Retrospectively registered.

**Supplementary Information:**

The online version contains supplementary material available at 10.1186/s12885-023-11739-9.

## Introduction

Brain metastases (BMs) are the most common intracranial malignancy, with an incidence of approximately 8.3–14.3 per 100,000 people [1, 2]. Approximately 10–30% of cancer patients develop brain metastases during the course of the disease [3, 4]. For patients with malignant tumours, once BMs are observed, the disease is considered to have progressed to an advanced stage, and is frequently linked with a poor prognosis [2]. The median overall survival (OS) of patients with BMs receiving only symptomatic relief treatments is 1–2 months [5], and approximately 20–30% of patients die due to poor intracranial tumour control [6].

Currently, the primary treatments for brain metastases are surgery, radiation therapy, chemotherapy, targeted therapy, and immunotherapy [7]. For decades, whole brain radiation therapy (WBRT) has been utilised as the standard treatment option to relieve most patients’ symptoms and prolong their survival by several months [8, 9]. This is especially true for patients who are not suitable candidates for general surgery or stereotactic radiosurgery (SRS) [10]. According to the results of several studies, however, WBRT provides poor control of existing metastases [11, 12]. Therefore, WBRT plus a lesion-targeting radiation boost has been introduced as an effective strategy for improved tumour control [13–19]. This included three boost schemes: WBRT + SRS, WBRT with a simultaneous integrated boost (WBRT + SIB), and WBRT with sequential integrated boost (WBRT + SEB). Compared with others, WBRT + SIB does not prolong the overall duration of radiation, and requires only one radiation treatment plan [14]. Having a single treatment plan helps us confirm the dose to the organs at risk (OARs), and further reduces radiation therapy-related toxicities. Moreover, WBRT + SIB could be used to treat large-diameter tumours, for which SRS is not an option [20]. Studies on WBRT + SIB are still limited, although several small-scale retrospective studies have reported potential survival benefits for WBRT + SIB over WBRT [19, 21, 22]. WBRT + SIB, however, has not been extensively analysed in selected populations, such as small cell lung cancer (SCLC) patients, and research on toxicities and dose selection remains limited. The present study, therefore, aimed to evaluate whether WBRT + SIB provides any therapeutic benefit over WBRT alone.

## Materials and methods

### Patient selection

A total of 499 patients who underwent brain radiotherapy in our department between January 2012 and June 2021 were identified and retrospectively analysed. The treatment plans for all enrolled patients were comprehensively determined by physicians based on recommendations from National Comprehensive Cancer Network (NCCN) guidelines [23], the number and location of brain metastases, and the general condition of the patients. In this study, we applied consistent inclusion and exclusion criteria for patient selection. The patient inclusion criteria were as follows: 1) primary solid tumour confirmed pathologically, with contrast-enhanced computed tomography (CT)- or magnetic resonance imaging (MRI)-verified BMs (no limitation on the number of BMs); 2) age ≥ 18 years; and 3) undergoing brain radiation. Prior surgery for BMs was allowed only if the patient experienced a postoperative relapse. The exclusion criteria were as follows: 1) undergoing either SRS or stereotactic radiation therapy (SRT); 2) undergoing prophylactic cranial irradiation (PCI); 3) undergoing two-dimensional radiation therapy; 4) previous intracranial radiation; and 5) inadequate follow-up imaging for at least 3 months. The following factors from each patient were analysed: age, sex, smoking status, drinking status, Karnofsky Performance Scale (KPS), pathological tumour type, number of BMs, extracranial disease status, extracranial metastases, presence or absence of meningeal or liver metastases, surgery before radiation, systemic treatment, radiotherapy dosages, dose fraction regimens, biologically effective dose (BED), radiotherapy technique, gross target volume (GTV), clinical target volume (CTV), planning gross target volume (PGTV), planning target volume (PTV), sum of the longest diameter of the BMs, treatment-related toxicities and date of initial treatment, BM diagnosis, first day of radiation treatment, intracranial progression, and death or final follow-up visit. Furthermore, a prognostic index, the Graded Prognostic Assessment (GPA), was calculated for each patient, which was the sum of scores (0, 0.5, and 1.0) for four factors: age, KPS, extracranial metastases, and number of BMs [24].

### Radiotherapy strategy

To ensure reproducibility, all patients were placed in a supine position, in a custom-made thermoplastic mask, and underwent a simulation CT scan of the entire head, using a 3- or 5-mm slice thickness. The CT scans were then merged with available contrast-enhanced T1-weighted MRI sequences. The target delineation criteria for both groups were the same: GTV was defined as brain metastases; CTV was defined as the whole brain tissue; PGTV was defined as GTV plus 5-mm margins; and PTV was defined as CTV plus 5-mm margins. The OARs were also delineated, primarily including the brainstem, eyes, lenses, optic chiasma, and optic nerves. Radiotherapy was delivered using intensity-modulated radiation therapy (IMRT), volumetric-modulated arc therapy (VMAT), or three-dimensional conformal radiotherapy (3D-CRT). For patients who underwent WBRT + SIB, the prescribed dose to the PTV was 25–37.5 Gy in 10–20 fractions, and the simultaneous boost to the PGTV was 35–52.5 Gy in 10–20 fractions (5 fractions per week). The dose for WBRT alone was 30 Gy in 10 fractions to the PTV (5 fractions per week) without further dose escalation.

### Follow-up

Follow-up examinations included brain MRI or CT, physical examinations, and toxicity evaluations conducted at least every 3 months for the first 2 years, and every 6 months thereafter, according to clinical protocol. Responses to treatment were assessed by experienced radiologists using the Response Assessment in Neuro-Oncology Brain Metastases (RANO-BM) criteria [25]. Toxicity was scored using the Common Toxicity Criteria Adverse Events version 5.0 (CTCAE v.5.0).

### Study endpoints

The primary endpoints of the present study were intracranial progression-free survival (PFS) and local tumour control (LTC). Intracranial PFS was defined as the time from the first day of radiation exposure to intracranial progression (including both the progression of irradiated BMs and the appearance of new lesions), death from any cause, or the final follow-up visit. Intracranial LTC was evaluated from the first day of radiation exposure to intracranial local tumour progression (only including progression of irradiated BMs), death, or the final follow-up visit. Progression was defined as a relative increase of the sum of the longest diameters of the BMs of ≥20% from the baseline, according to the RANO-BM criteria. Treatment-related modifications, such as radiation necrosis, were not regarded as local failures.

The secondary endpoints of the present study were OS and radiation-related toxicity. OS was defined as the time from the first day of radiation exposure to death or final follow-up. Radiation-induced brain injury was diagnosed by experienced radiologists using MRI and magnetic resonance spectroscopy (MRS). The survival and toxicity data were obtained from hospital records or direct correspondence with the referring physician or relatives of patients.

### Statistical analysis

Descriptive characteristics were analysed using the chi-squared and Fisher’s exact tests, and continuous variables were evaluated using the t-test and Mann-Whitney U test. A propensity score matching (PSM) analysis was conducted with logistic regression considering the following: sex, GPA scores, primary tumour type, and number of BMs. We employed the Kaplan-Meier method, log-rank test, and landmark survival analyses to compare intracranial PFS, LTC, and OS among different groups. Specifically, Competing Risk analyses, accounting for deaths unrelated to intracranial tumour progression as competing risks, were particularly utilized to assess intracranial PFS and LTC across the groups. Univariate and multivariate analyses were performed using the Cox regression models. Additionally, the factors with *P* < 0.2 in univariate analysis were included in the multivariate analysis. A receiver operating characteristic (ROC) curve was used to assess toxicity. PSM analysis and stratified analysis were utilized to control and adjust for potential allocation bias, enhancing the internal validity of this study. SPSS (version 26.0), R (version 4.1.2), RStudio, and GraphPad Prism (version 9.3.0) were used for the analyses. All *P*-values were two-sided, with *P* < 0.05. considered statistically significant.

## Results

### Patients

A total of 165 patients were included for analysis in the present study: 82 in the WBRT + SIB group, and 83 in the WBRT group. The baseline characteristics of the two groups are shown in Table 1. Notably, the WBRT group had a higher proportion of breast cancer patients (16.9% vs. 1.2%), leading to imbalances in sex and primary tumour type (*P* = 0.004 and *P* < 0.001, respectively). Furthermore, the WBRT + SIB group exhibited higher GPA scores compared to the WBRT group (69.6% with GPA ≥ 2 vs. 51.8%; *P* = 0.017). All other characteristics were well-balanced between the two groups. Subsequently, after conducting a PSM analysis, the patient characteristics were re-evaluated and presented in Table 1, demonstrating that all characteristics were well-balanced. Details concerning radiotherapy-related characteristics can be found in Supplementary Table 1.

**Table 1:** Patient characteristics

Characteristics	Before PSM			After PSM		
	WBRT + SIB (*n* = 82)	WBRT (*n* = 83)	*P* value	WBRT + SIB (*n* = 52)	WBRT (*n* = 52)	*P* value
Age, median(range), years	60(27–78)	59 (34–80)	0.634	60 (27–74)	61 (34–80)	0.974
Sex						
Male	61 (74.4%)	44 (53.0%)	0.004^*^	32 (61.5%)	35 (67.3%)	0.539
Female	21 (25.6%)	39 (47.0%)		20 (38.5%)	17 (32.7%)	
KPS						
90–100	26 (31.7%)	28 (33.7%)	0.876	12 (23.1%)	16 (30.8%)	0.751
70–80	54 (65.9%)	52 (62.7%)		39 (75.0%)	35 (67.3%)	
< 70	2 (2.4%)	3 (3.6%)		1 (1.9%)	1 (1.9%)	
Primary tumour						
NSCLC	39 (47.6%)	40 (48.2%)	< 0.001^*^	28 (47.6%)	25 (48.2%)	0.675
SCLC	35 (42.7%)	28 (33.7%)		20 (42.7%)	25 (33.7%)	
Breast cancer	1 (1.2%)	14 (16.9%)		1 (1.9%)	1 (1.9%)	
Other cancer	7 (8.5%)	1 (1.2%)		3 (5.8%)	1 (1.9%)	
GPA						
3.0 ≤ GPA < 4.0	18 (22.0%)	10 (12.0%)	0.017^*^	3 (5.8%)	8 (15.4%)	0.251
2.0 ≤ GPA < 3.0	39 (47.6%)	33 (39.8%)		18 (34.6%)	21 (40.4%)	
1.0 ≤ GPA < 2.0	22 (26.8%)	26 (31.3%)		24 (46.2%)	16 (30.8%)	
GPA < 1.0	3 (3.7%)	14 (16.9%)		7 (13.5%)	7 (13.5%)	
Extracranial metastases						
Yes	30 (36.6%)	40 (48.2%)	0.132	21(40.4%)	20(38.5%)	0.841
No	52 (63.4%)	43 (51.8%)		31 (59.6%)	32 (61.5%)	
Extracranial disease						
Stable	57 (69.5%)	61 (73.5%)	0.571	34 (65.4%)	37 (71.2%)	0.527
Active	25 (30.5%)	22 (26.5%)		18 (34.6%)	15 (28.8%)	
Brain surgery before RT						
Yes	4 (4.9%)	3 (3.6%)	0.987	3 (5.8%)	2 (3.8%)	> 0.999
No	78 (95.1%)	80 (96.4%)		49 (94.2%)	50 (96.2%)	
Sum of longest diameters of BM, mean (range), cm	3.6 (0.6–11.0)	4.4 (1.0–15.3)	0.291	4.0 (0.6–11.0)	4.0 (1.0–12.8)	0.782
Targeted therapy						
Yes	16 (19.5%)	26 (31.3%)	0.082	13 (25.0%)	15 (28.8%)	0.658
No	66 (80.5%)	57 (68.7%)		39 (75.0%)	37 (71.1%)	
Immunotherapy						
Yes	8 (9.8%)	5 (6.0%)	0.374	6 (11.5%)	5(9.6%)	0.750
No	74 (90.2%)	78 (94.0%)		46 (88.5%)	47 (90.4%)	
Meningeal metastasis						
Yes	5 (6.1%)	2 (2.4%)	0.430	3 (5.8%)	0 (0.0%)	0.241
No	77 (93.9%)	81 (97.6%)		49 (94.2%)	52 (100.0%)	
Salvage therapy^†^						
Yes	6 (7.3%)	13 (15.7%)	0.093	5 (9.6%)	10 (19.2%)	0.163
No	76 (92.7%)	70 (84.3%)		47 (90.4%)	42 (80.8%)	

Abbreviations: *PSM* propensity score matching, *WBRT* whole-brain radiation therapy, *SIB* simultaneous integrated boost, *KPS* Karnofsky Performance Status, *NSCLC* non-small cell lung cancer, *SCLC* small cell lung cancer, *GPA* graded prognostic assessment, *RT* radiotherapy, *BM* brain metastasis. *P < 0.05. †Salvage therapy refers to patients undergoing further brain radiation or brain surgery following the progression of brain tumours

### Intracranial PFS, LTC, and OS

**Fig. 1:**
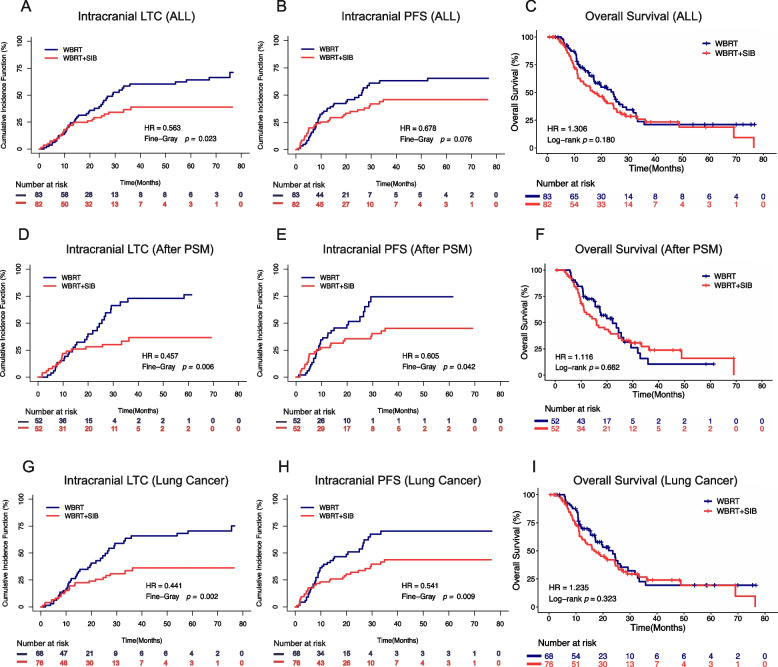
Comparison of intracranial local tumour control (LTC) (**A**), intracranial progression-free survival (PFS) (**B**), and overall survival (OS) (**C**) between whole brain radiation therapy with a simultaneous integrated boost (WBRT + SIB) and whole brain radiation therapy (WBRT) in all patients using competing risk analyses. Comparison of intracranial LTC (**D**), intracranial PFS (**E**), and OS (**F**) between WBRT + SIB and WBRT after propensity score matching (PSM) using competing risk analyses. Comparison of intracranial LTC (**G**), intracranial PFS (H), and OS (**I**) between WBRT + SIB and WBRT in the lung cancer subgroup using competing risk analyses

For all patients, the cumulative incidence of local intracranial progression was significantly lower in the WBRT + SIB group, leading to a significantly longer intracranial local tumour control (LTC) period (*P* = 0.023; Fig. 1A). Similarly, WBRT + SIB also slightly prolonged patients’ intracranial progression-free survival (PFS), although not reaching statistical significance (*P* = 0.076; Fig. 1B). However, there were no significant benefits in overall survival (OS) for the WBRT + SIB group compared to WBRT alone (*P* = 0.180; Fig. 1C). Of note, the survival outcomes after PSM analysis were consistent with the aforementioned results, showing that the WBRT + SIB group achieved significantly longer intracranial LTC and PFS (*P* = 0.006 and 0.042, respectively; Figs. 1D, E), but still no improvement in overall survival (*P* = 0.662; Fig. 1F).

Then we conducted additional survival analyses in the lung cancer subgroup, which included 68 patients receiving WBRT and 76 patients receiving WBRT + SIB. The results indicated that the WBRT + SIB group had a dramatically improved intracranial LTC and PFS compared with the WBRT group (*P* = 0.002 and 0.009, respectively; Figs. 1G, H). There was no significant difference, however, in terms of OS (*P* = 0.323; Fig. 1I).

Besides, the intracranial LTC and PFS curves presented in Fig. 1A, B, D, E, G, and H were further analysed using the Kaplan-Meier method, as demonstrated in Supplementary Fig. 1. The outcomes remained consistent with the earlier results.

### Predictive factors of intracranial PFS, LTC, and OS

As shown in Supplementary Table 2, univariate analysis revealed that treatment method, extracranial disease control, and GPA score had substantial impacts on intracranial PFS in the lung cancer subgroup (*P* = 0.021, 0.172, and 0.029, respectively). Furthermore, multivariate analysis indicted that treatment with WBRT+SIB was an independent influencing factor strongly associated with increased intracranial PFS compared to WBRT alone (*P* = 0.026).

Similarly, we further examined the factors that influenced LTC and OS in the lung cancer subgroup, and found that treatment methods and GPA scores were the major contributing factors to intracranial LTC (*P* = 0.044 and 0.054, respectively; Supplementary Table 3). Multivariate analysis of OS revealed that pathological type non-small cell lung cancer (NSCLC), well-controlled extracranial disease, and higher GPA were associated with increased OS (*P* = 0.004, 0.004, and 0.02, respectively; Supplementary Table 4).

### Stratified analysis

To minimise the impact of other predictive factors and the difference in baseline GPA scores, stratified analyses was performed by dividing lung cancer patients with BMs into groups based on GPA scores, extracranial disease control, and pathological tumour type. The relevant information regarding the subgroups is presented in Supplementary Table 5.

**Fig. 2:**
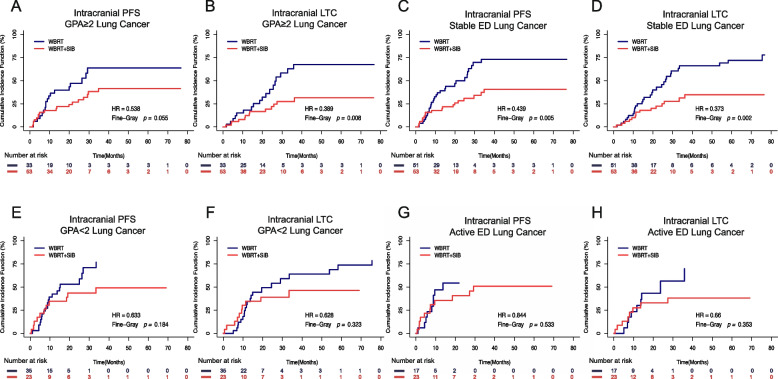
Comparison of intracranial progression-free survival (PFS) (**A**) and local tumour control (LTC) (**B**) for WBRT and WBRT + SIB in lung cancer patients with a graded prognostic assessment (GPA) score ≥ 2 using competing risk analyses. Comparison of intracranial PFS (**C**) and LTC (**D**) for WBRT and WBRT + SIB in lung cancer patients with stable extracranial disease (ED) using competing risk analyses. Comparison of intracranial PFS (**E**) and LTC (**F**) for WBRT and WBRT + SIB in lung cancer patients with a GPA < 2 using competing risk analyses. Comparison of intracranial PFS (**G**) and LTC (**H**) for WBRT and WBRT + SIB in lung cancer patients with active ED using competing risk analyses

The results of the stratified analysis indicated that WBRT + SIB dramatically increased intracranial PFS and LTC in patients with a GPA ≥ 2 or stable extracranial disease, when compared to WBRT alone (*P* = 0.055, 0.008, 0.005, and 0.002, respectively; Figs. 2A, B, C, D), whereas patients with a GPA < 2 or active extracranial disease saw no benefit from WBRT + SIB vs. WBRT alone (*P* = 0.184, 0.323, 0.533, and 0.353, respectively; Figs. 2E, F, G, H). All the results of the stratified analysis were reconfirmed through the Kaplan-Meier method, as illustrated in Supplementary Fig. 2.

**Fig. 3:**
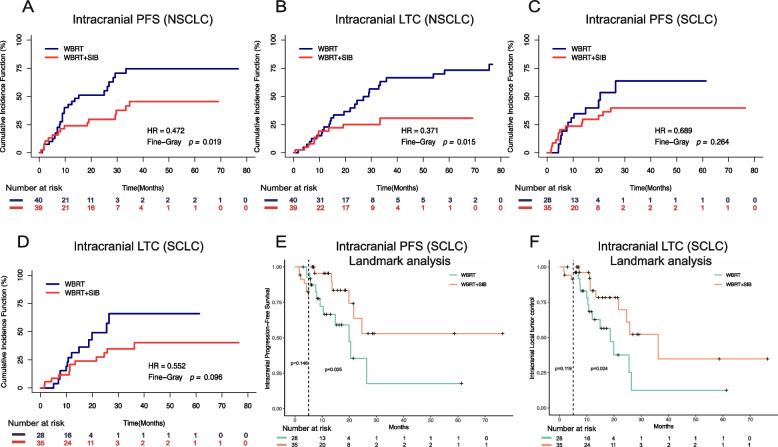
Comparison of intracranial progression-free survival (PFS) (**A**) and local tumour control (LTC) (**B**) for WBRT and WBRT + SIB in non-small cell lung cancer patients (NSCLC) using competing risk analyses. Comparison of intracranial PFS (**C**) and LTC (**D**) for WBRT and WBRT + SIB in small cell lung cancer (SCLC) patients using competing risk analyses. Landmark analysis of intracranial PFS (**E**) and LTC (**F**) in SCLC patients receiving WBRT + SIB and WBRT (cutoff time = 5 months)

Additionally, NSCLC patients who underwent treatment with WBRT + SIB exhibited notable improvements in both intracranial PFS and LTC (*P* = 0.019 and 0.015, respectively; Figs. 3A, B). For small cell lung cancer (SCLC) patients, however, we found no remarkable differences in intracranial PFS or LTC between the two groups (*P* = 0.264 and 0.096, respectively; Figs. 3C, D). Consistent results were obtained through the Kaplan-Meier method, as depicted in Supplementary Fig. 3. Then we further conducted landmark survival analysis, we did find that treatment with WBRT + SIB significantly improved the long-term intracranial PFS and LTC (> 5 months) of SCLC patients (*P* = 0.025 and 0.024, respectively; Figs. 3E, F), although no such improvement was seen in short-term survival (*P* = 0.146 and 0.119, respectively; Fig. 3E, F).

### Toxicity

As shown in Table 2, the majority of the patients included in the present study experienced radiotherapy-related toxicities, which primarily involved nausea, vomiting, dizziness, headache, radiation-induced brain injury, or other central nervous system (CNS) symptoms. The incidence of grade 3 or worse adverse events in the WBRT + SIB and WBRT groups was 0 vs. 2.4% (*P* = 0.497) for headache, 1.2 vs. 1.2% (*P* > 0.999) for dizziness, 1.2 vs. 0% (*P* = 0.497) for radiation-induced brain injury, and 0 vs. 2.4% (*P* = 0.497) for leukopenia, respectively.

**Table 2:** Adverse events

Adverse events	WBRT + SIB (*n* = 82)		WBRT (*n* = 83)	
	Grade 1 or 2, n (%)	Grade 3, n (%)	Grade 1 or 2, n (%)	Grade 3, n (%)
Fatigue	11(13)	0(0)	9(11)	0(0)
Headache	21(26)	0(0)	20(24)	2(2)
Dizziness	13(16)	1(1)	21(25)	1(1)
Nausea	11(13)	0(0)	16(19)	0(0)
Vomiting	5(6)	0(0)	9(11)	0(0)
Fever	2(2)	0(0)	1(1)	0(0)
Leukopenia	8(10)	0(0)	5(6)	2(2)
Thrombocytopenia	0(0)	0(0)	2(2)	0(0)
Epilepsy	3(4)	0(0)	1(1)	0(0)
Radiation dermatitis	1(1)	0(0)	0(0)	0(0)
Consciousness disorder	0(0)	0(0)	1(1)	0(0)
Cephalic and facial edema	0(0)	0(0)	1(1)	0(0)
Supraventricular tachycardia	0(0)	0(0)	1(1)	0(0)
Hypokalemia	0(0)	0(0)	1(1)	0(0)
Radiation-induced brain injury	7(9)	1(1)	2(2)	0(0)

Abbreviations: *WBRT* whole-brain radiation therapy, *SIB* simultaneous integrated boost

**Fig. 4:**
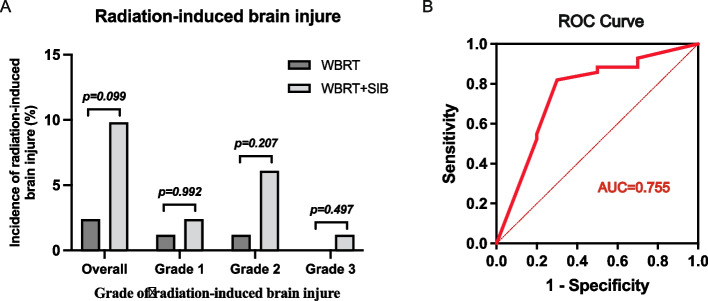
Incidence of radiation-induced brain injury for WBRT + SIB and WBRT groups in all patients (**A**). Receiver operating characteristic (ROC) curves showed the correlation between biologically effective doses given to brain metastases and the incidence of radiation-induced brain injury (**B**)

Additionally, when evaluating the incidence of radiation-induced brain injury, there were 8 cases (9.8%), including 1 case of grade 3 radiation-induced brain injury, in the WBRT + SIB group, whereas the WBRT group reported only 2 cases (2.4%), neither of which were grade 3. The incidence of radiation-induced brain injury was higher in the WBRT + SIB group for all grades (grades 1 to 3, *P* = 0.992, 0.207, and 0.497, respectively; Fig. 4A), and the same was seen for overall risk (*P* = 0.099; Fig. 4A). We further explored the association between the risk of radiation-induced brain injury and the booster dosage administered in the WBRT + SIB group, and found that the risk rose with an increased dosage (area under the curve [AUC] = 0.755; Fig. 4B). Moreover, to determine the optimal dose for WBRT + SIB therapy, we separated the patients into two subgroups: low-dose (BED ≤56 Gy) and high-dose (BED > 56 Gy), and found that low-dose WBRT + SIB had a lower incidence of radiation-induced brain injury than high-dose WBRT + SIB (*P* = 0.020). Additionally, no significant difference was found when compared with WBRT alone (*P* > 0.999).

## Discussion

In the present study, we found that when utilised as the first-line treatment for BMs, WBRT + SIB resulted in better intracranial tumour control than WBRT alone. Furthermore, low-dose WBRT + SIB (BED ≤56 Gy) was preferred to high-dose WBRT + SIB because of the association between the boost dosage and the incidence of radiation-induced brain injury.

Although WBRT is still considered the standard treatment option for BMs, the median intracranial PFS after such treatment among patients with multiple BMs is only approximately 5–7 months [18, 26]. In the present study, we discovered that WBRT + SIB could prolong the intracranial LTC of existing metastases, which was also reflected in the slightly improved intracranial PFS. And after PSM analysis, the survival outcomes were consistent with these results, showing that the WBRT + SIB group achieved significantly longer intracranial LTC and PFS without baseline differences between the two groups. Moreover, to minimise the impact of baseline differences in sex and primary tumour type, further analysis was performed in the lung cancer subgroup, in which we found that lung cancer patients treated with WBRT + SIB demonstrated greater improvements in intracranial PFS and LTC. Univariate and multivariate analyses also indicated that the treatment method (WBRT vs. WBRT + SIB) was an independent prognostic factor for patients’ intracranial PFS and LTC.

The results of the present study were consistent with those of Popp et al. [18], who evaluated 124 patients with at least 4 BMs from various primary tumour types (excluding SCLC) treated with an average of 35 Gy WBRT or 30 Gy WBRT with 42 or 51 Gy SIB in 12 fractions. They found that WBRT + SIB was associated with improved intracranial PFS and OS than WBRT. The results of the present study, however, did not indicate any improvement in survival benefits in the WBRT + SIB group, even after PSM analysis, which might be attributed to the following factors: 1) more patients experienced meningeal metastasis in the WBRT + SIB group than the WBRT group (5.8 vs. 0%); 2) there was a lower proportion of targeted therapy in the WBRT + SIB group than the WBRT group (25.0 vs. 28.8%); and 3) there was a lower incidence of salvage therapy (brain radiation or surgery, 9.6 vs. 19.2%) in the WBRT + SIB group compared to the WBRT group. In fact, the benefit of WBRT + SIB treatment on survival remains controversial. In line with our results, several studies have shown that the dose escalation strategy did not improve patient survival compared with WBRT alone [15, 27, 28].

On the other hand, the existence of brain metastases had a profound impact on both the quality of life (QOL) and neurological status of patients [29]. Research suggested that employing WBRT-alone for brain metastases offers limited benefits in terms of both QOL and neurological status [30–34]. Conversely, dose escalation strategies like SRS demonstrated clear advantages [12]. Notably, our study highlighted that WBRT+SIB treatment significantly enhances intracranial local tumour control, effectively delaying brain tumour progression and recurrence in patients with brain metastases. This implies that, despite the absence of an OS advantage compared to WBRT-alone treatment, WBRT+SIB treatment may still hold potential benefits in terms of enhancing QOL and neurological status. However, owing to the retrospective nature of this study, we have been unable to access relevant information for analysis. Further research is imperative to substantiate these aspects.

Univariate analysis in the present study revealed that both GPA and extracranial disease contributed to the control of intracranial tumours. In previous studies, they were recognised as independent influencing factors for intracranial PFS, which is consistent with the findings of the present study [15, 27]. Additionally, due to the retrospective nature of this study, a certain degree of allocation bias may exist, mainly arising from decisions made by physicians or patients. Consequently, the WBRT + SIB group in this study exhibited higher baseline GPA scores than the WBRT group. To minimize the impact of these factors, except for the previous PSM analysis, we also conducted a stratified analysis within the lung cancer subgroup. The results presented patients with a GPA ≥ 2 and stable extracranial disease had substantially improved intracranial PFS and LTC following WBRT + SIB treatment compared to WBRT alone, while patients with a GPA < 2 and active extracranial disease exhibited no therapeutic benefit. This implies that, despite differences in baseline characteristics, WBRT + SIB treatment provided a clear advantage in terms of intracranial tumour local control, especially in the population with high GPA scores and stable extracranial disease. For patients with a GPA < 2 and active extracranial disease, the stratified analysis did not reveal a clear therapeutic benefit. We hypothesize that this could be attributed to the fact that patients with poor prognostic factors, such as low KPS, multiple BMs, or extracranial metastases are more likely to have shorter survival times [24, 35], and the advantage of WBRT + SIB over WBRT in terms of intracranial tumour local control is not obvious in that limited time. These findings further support the use of WBRT + SIB in patients with improved baseline conditions.

On the other hand, we first reported SCLC patients with BMs who underwent WBRT + SIB had improved intracranial PFS and LTC at long-term survival (> 5 months). Available research on WBRT + SIB in SCLC patients is limited, with only one small retrospective study demonstrating that WBRT + SIB prolonged OS compared to WBRT alone [22]. Although SCLC patients have a high risk of BMs, which are rarely isolated and characterised by early intracranial dissemination [22, 36], the findings of the present study suggest the use of WBRT + SIB treatment in SCLC patients for better long-term intracranial tumour control. Additionally, added benefits of LTC were also seen in NSCLC patients following WBRT + SIB treatment, consistent with earlier studies [27].

In terms of adverse events, there was no significant difference in the occurrence of grade 3 or higher toxicities between the two groups; however, we discovered that radiation-induced brain injury occurred more frequently in the WBRT + SIB group (overall, 9.8 vs. 2.4%). Furthermore, we noticed that the incidence of radiation-induced brain injury increased as the BED administered increased. One study reported a radiation necrosis incidence of approximately 3.2% following WBRT + SIB treatment [18], which was slightly lower than what we observed in the present study. It may be attributed to the different definitions between radiation necrosis and radiation-induced brain injury, as well as the utilization of distinct diagnostic methods in the study. The confirmation of radiation necrosis relied on pathological examination, whereas we diagnosed radiation-induced brain injury using MRI and MRS. [37–39]. Therefore, the findings of the present study indicate that treatment with WBRT + SIB, particularly high-dose, may increase the risk of radiation-induced brain injury. And this risk was found to be significantly lower following low-dose (BED ≤56 Gy) WBRT + SIB than high-dose WBRT + SIB, and was equivalent to that of WBRT alone. Moreover, using both competing risk analyses and the Kaplan-Meier approach, we found no difference in intracranial tumour control between the low- and high-dose WBRT + SIB groups. (*P* = 0.654, 0.471, 0.532, and 0.365, respectively; Supplementary Figs. 4A, B, C, D). Therefore, the results of the present study confirmed that low-dose WBRT + SIB treatment could allow for better intracranial tumour control while simultaneously minimising the risk of radiation-induced brain injury. Based on our findings, we recommend 30 Gy in 10 fractions for PTV, and 40 Gy in 10 fractions for PGTV.

The present study had several limitations. Firstly, due to the retrospective nature of this study, researchers were unable to randomly control the allocation process of participants, leading to potential allocation bias, such as the imbalance in baseline GPA scores between the two treatment groups. Despite significant efforts to mitigate this bias through methods like PSM analysis and stratified analyses, the study results may still be influenced. Second, there was no evaluation of the patients’ quality of life and neurological status, due to the absence of corresponding data. Third, because this was a small retrospective study with inherent biases, the findings should be verified in future prospective studies.

## Conclusions

In conclusion, treatment with WBRT + SIB may improve intracranial tumour control in patients with BMs compared to WBRT alone, particularly in the lung cancer subgroup. Patients with higher GPA scores and well-controlled extracranial disease undergoing WBRT + SIB had improved intracranial PFS and LTC than those undergoing WBRT alone. Moreover, the use of WBRT + SIB also led to better long-term intracranial control in SCLC patients. The results of the present study demonstrated that treatment with WBRT + SIB might increase the risk of radiation-induced brain injury, with that risk being proportional to the boost dosage. Low-dose WBRT + SIB (BED ≤56 Gy) showed good tumour control and an acceptable risk of radiation-induced brain injury; therefore, low-dose WBRT + SIB should be considered for patients with BMs, although further prospective studies are needed to validate our findings.

### Supplementary Information

**Additional file 1. **Supplementary Table 1 - 5.

**Additional file 2.** Supplementary Fig. 1 Comparison of intracranial local tumour control (LTC) (A) and progression-free survival (PFS) (B) between whole brain radiation therapy with a simultaneous integrated boost (WBRT + SIB) and whole brain radiation therapy (WBRT) in all patients using Kaplan-Meier method. Comparison of intracranial LTC (C) and PFS (D) between WBRT + SIB and WBRT after propensity score matching (PSM) using Kaplan-Meier method. Comparison of intracranial LTC (E) and PFS (F) between WBRT + SIB and WBRT in the lung cancer subgroup using Kaplan-Meier method.

**Additional file 3.** Supplementary Fig. 2 Comparison of intracranial progression-free survival (PFS) (A) and local tumour control (LTC) (B) for WBRT and WBRT + SIB in lung cancer patients with a graded prognostic assessment (GPA) score ≥ 2 using Kaplan-Meier method. Comparison of intracranial PFS (C) and LTC (D) for WBRT and WBRT + SIB in lung cancer patients with stable extracranial disease (ED) using Kaplan-Meier method. Comparison of intracranial PFS (E) and LTC (F) for WBRT and WBRT + SIB in lung cancer patients with a GPA < 2 using Kaplan-Meier method. Comparison of intracranial PFS (G) and LTC (H) for WBRT and WBRT + SIB in lung cancer patients with active ED using Kaplan-Meier method.

**Additional file 4.** Supplementary Fig. 3 Comparison of intracranial progression-free survival (PFS) (A) and local tumour control (LTC) (B) for WBRT and WBRT + SIB in non-small cell lung cancer patients (NSCLC) using Kaplan-Meier method. Comparison of intracranial PFS (C) and LTC (D) for WBRT and WBRT + SIB in small cell lung cancer (SCLC) patients using Kaplan-Meier method.

**Additional file 5.** Supplementary Fig. 4 Comparison of intracranial progression-free survival (PFS) (A) and local tumour control (LTC) (B) for low-dose and high-dose WBRT + SIB group using competing risk analyses. Comparison of intracranial PFS (C) and LTC (D) for low-dose and high-dose WBRT + SIB group using Kaplan-Meier method.

## Data Availability

The datasets used and/or analysed during the current study are available from the corresponding author on reasonable request.

## Abbreviations

BMs: brain metastases
WBRT + SIB: whole brain radiation therapy with a simultaneous integrated boost
WBRT: whole brain radiation therapy
PFS: progression-free survival
LTC: local tumour control
OS: overall survival
BED: biologically effective dose
SRS: stereotactic radiosurgery
WBRT + SEB: whole brain radiation therapy with sequential integrated boost
OARs: organs at risk
SCLC: small cell lung cancer
NCCN: National Comprehensive Cancer Network
CT: computed tomography
MRI: magnetic resonance imaging
SRT: stereotactic radiation therapy
PCI: prophylactic cranial irradiation
KPS: karnofsky performance scale
GTV: gross target volume
CTV: clinical target volume
PGTV: planning gross target volume
PTV: planning target volume
GPA: graded prognostic assessment
IMRT: intensity-modulated radiation therapy
VMAT: volumetric-modulated arc therapy
3D-CRT: three-dimensional conformal radiotherapy
RANO-BM: neuro-oncology brain metastases
CTCAE v.5.0: common toxicity criteria adverse events version 5.0
MRS: magnetic resonance spectroscopy
PSM: propensity score matching
ROC: receiver operating characteristic
NSCLC: non-small cell lung cancer
CNS: central nervous system
QOL: quality of life.
