# Supplementary material for: Adding simultaneous integrated boost to whole brain radiation therapy improved intracranial tumour control and minimize radiation-induced brain injury risk for the treatment of brain metastases
Source: BMC Cancer. 2023 Dec 16;23:1240. doi: 10.1186/s12885-023-11739-9 (PMC10724957; doi:10.1186/s12885-023-11739-9)
Supplement: Supplementary file 1 — Additional file 1. Supplementary Table 1 - 5. [file 12885_2023_11739_MOESM1_ESM.docx]

**Supplementary Table 1. Radiotherapy characteristics**

| **Characteristics** | **WBRT+SIB(n=82)** | **WBRT(n=83)** |
| --- | --- | --- |
| **GTV, mean (range), cm^3^** | 12.5(0.5-65.3) | - |
| **PGTV, mean (range), cm^3^** | 30.3(0.9-133.3) | - |
| **CTV, mean (range), cm^3^** | 1358.0(1058.2-1598.1) | 1336.1(1095.4-1685.2) |
| **PTV, mean (range), cm^3^** | 1641.0(1088.7-2029.3) | 1626.0(1306.4-2085.9) |
| **Boosting dose of BM, Gy** |  |  |
| 30 | - | 83(100%) |
| 35 | 4(4.9%) | - |
| 40 | 43(52.4%) | - |
| 42 | 10(12.2%) | - |
| 44.4 | 1(1.2%) | - |
| 45 | 4(4.9%) | - |
| 45.6 | 1(1.2%) | - |
| 48 | 1(1.2%) | - |
| 49 | 2(2.4%) | - |
| 50 | 15(1.2%) | - |
| 52.5 | 1(1.2%) | - |
| **BED, mean (range), Gy** | 59.9(47.3-75.0) | 39.0 |

Abbreviations: WBRT, whole-brain radiation therapy; SIB, simultaneous integrated boost; GTV, gross target volume; PGTV, planning gross target volume; CTV, clinical target volume; PTV, planning target volume; BM, brain metastasis; BED, biologically effective dose.

**Supplementary Table 2. Univariate and multivariate survival analyses on intracranial progression-free survival in lung cancer patients.**

|  | **Intracranial progression-free survival** | | | | | | |
| --- | --- | --- | --- | --- | --- | --- | --- |
|  | **Univariate analysis** | | |  | **Multivariate analysis** | | |
| **Clinical characteristics** | **HR** | **95% CI** | ***P value*** |  | **HR** | **95% CI** | ***P value*** |
| **Treatment** |  |  |  |  |  |  |  |
| WBRT vs. WBRT+SIB | 0.563 | (0.346-0.916) | 0.021^*^ |  | 0.565 | (0.341-0.935) | 0.026^*^ |
| **Pathology** |  |  |  |  |  |  |  |
| SCLC vs. NSCLC | 0.988 | (0.601-1.624) | 0.962 |  |  |  |  |
| **Extracranial disease** |  |  |  |  |  |  |  |
| Stable vs. Active | 0.694 | (0.410-1.173) | 0.172 |  | 0.648 | (0.378-1.111) | 0.115 |
| **GPA** |  |  |  |  |  |  |  |
| High vs. Low | 0.584 | (0.361-0.946) | 0.029^*^ |  | 0.658 | (0.404-1.074) | 0.094 |
| **Concurrent chemotherapy** |  |  |  |  |  |  |  |
| Yes vs. No | 0.971 | (0.352-2.676) | 0.954 |  |  |  |  |
| **Targeted therapy** |  |  |  |  |  |  |  |
| Yes vs. No | 1.032 | (0.620-1.716) | 0.904 |  |  |  |  |
| **Immunotherapy** |  |  |  |  |  |  |  |
| Yes vs. No | 1.115 | (0.549-2.263) | 0.763 |  |  |  |  |
| **Smoking** |  |  |  |  |  |  |  |
| Yes vs. No | 0.918 | (0.566-1.488) | 0.728 |  |  |  |  |
| **Drinking** |  |  |  |  |  |  |  |
| Yes vs. No | 0.906 | (0.541-1.516) | 0.706 |  |  |  |  |

Abbreviations: WBRT, whole-brain radiation therapy; SIB, simultaneous integrated boost; SCLC, small cell lung cancer; NSCLC, non-small cell lung cancer; GPA, graded prognostic assessment; HR, hazard ratio; CI, confidence interval. *P < 0.05.

**Supplementary Table 3. Univariate and multivariate survival analyses on local tumor control in lung cancer patients**

|  | **Local tumor control** | | | | | | |
| --- | --- | --- | --- | --- | --- | --- | --- |
|  | **Univariate analysis** | | |  | **Multivariate analysis** | | |
| **Clinical characteristics** | **HR** | **95% CI** | ***P value*** |  | **HR** | **95% CI** | ***P value*** |
| **Treatment** |  |  |  |  |  |  |  |
| WBRT vs. WBRT+SIB | 0.521 | (0.310-0.874) | 0.013^*^ |  | 0.580 | (0.341-0.986) | 0.044^*^ |
| **Pathology** |  |  |  |  |  |  |  |
| SCLC vs. NSCLC | 1.213 | (0.723-2.035) | 0.464 |  |  |  |  |
| **Extracranial disease** |  |  |  |  |  |  |  |
| Stable vs. Active | 0.709 | (0.404-1.245) | 0.231 |  |  |  |  |
| **GPA** |  |  |  |  |  |  |  |
| High vs. Low | 0.537 | (0.326-0.886) | 0.015^*^ |  | 0.605 | (0.363-1.009) | 0.054 |
| **Smoking** |  |  |  |  |  |  |  |
| Yes vs. No | 0.813 | (0.492-1.344) | 0.420 |  |  |  |  |
| **Drinking** |  |  |  |  |  |  |  |
| Yes vs. No | 0.855 | (0.500-1.461) | 0.565 |  |  |  |  |
| **Targeted therapy** |  |  |  |  |  |  |  |
| Yes vs. No | 1.004 | (0.588-1.714) | 0.988 |  |  |  |  |
| **Immunotherapy** |  |  |  |  |  |  |  |
| Yes vs. No | 1.098 | (0.518-2.327) | 0.807 |  |  |  |  |
| **Concurrent chemotherapy** |  |  |  |  |  |  |  |
| Yes vs. No | 0.969 | (0.351-2.676) | 0.951 |  |  |  |  |

Abbreviations: WBRT, whole-brain radiation therapy; SIB, simultaneous integrated boost; SCLC, small cell lung cancer; NSCLC, non-small cell lung cancer; GPA, graded prognostic assessment; HR, hazard ratio; CI, confidence interval. *P < 0.05.

**Supplementary Table 4. Univariate and multivariate survival analyses on overall survival in lung cancer patients**

|  | **Overall survival** | | | | | | |
| --- | --- | --- | --- | --- | --- | --- | --- |
|  | **Univariate analysis** | | |  | **Multivariate analysis** | | |
| **Clinical characteristics** | **HR** | **95% CI** | ***P value*** |  | **HR** | **95% CI** | ***P value*** |
| **Treatment** |  |  |  |  |  |  |  |
| WBRT vs. WBRT+SIB | 1.235 | (0.812-1.878) | 0.325 |  |  |  |  |
| **Pathology** |  |  |  |  |  |  |  |
| SCLC vs. NSCLC | 0.617 | (0.405-0.942) | 0.025^*^ |  | 0.460 | (0.270-0.783) | 0.004^*^ |
| **Extracranial disease** |  |  |  |  |  |  |  |
| Stable vs. Active | 0.607 | (0.386-0.952) | 0.030^*^ |  | 0.481 | (0.291-0.796) | 0.004^*^ |
| **GPA** |  |  |  |  |  |  |  |
| High vs. Low | 0.669 | (0.439-1.019) | 0.061 |  | 0.592 | (0.380-0.922) | 0.02^*^ |
| **Liver Metastases** |  |  |  |  |  |  |  |
| Yes vs. No | 2.409 | (1.200-4.837) | 0.013^*^ |  | 1.998 | (0.969-4.12) | 0.061 |
| **Meningeal metastasis** |  |  |  |  |  |  |  |
| Yes vs. No | 1.254 | (0.395-3.979) | 0.701 |  |  |  |  |
| **Targeted therapy** |  |  |  |  |  |  |  |
| Yes vs. No | 0.707 | (0.440-1.138) | 0.153 |  | 0.891 | (0.505-1.574) | 0.692 |
| **Immunotherapy** |  |  |  |  |  |  |  |
| Yes vs. No | 0.582 | (0.268-1.262) | 0.170 |  | 0.644 | (0.291-1.425) | 0.278 |
| **Smoking** |  |  |  |  |  |  |  |
| Yes vs. No | 1.173 | (0.758-1.814) | 0.474 |  |  |  |  |
| **Drinking** |  |  |  |  |  |  |  |
| Yes vs. No | 0.839 | (0.535-1.315) | 0.444 |  |  |  |  |

Abbreviations: WBRT, whole-brain radiation therapy; SIB, simultaneous integrated boost; SCLC, small cell lung cancer; NSCLC, non-small cell lung cancer; GPA, graded prognostic assessment; HR, hazard ratio; CI, confidence interval. *P < 0.05.

**Supplementary Table 5. Grouping information for stratified analysis**

| **Stratified Grouping** | **Number of Lung Cancer Patients** | |
| --- | --- | --- |
|  | **WBRT+SIB (n=76)** | **WBRT (n=68)** |
| **GPA score** |  |  |
| GPA< 2 | 23 | 35 |
| GPA≥ 2 | 53 | 33 |
| **Extracranial disease control situation** |  |  |
| Active extracranial disease | 23 | 17 |
| Stable extracranial disease | 53 | 51 |
| **Pathological type^†^** |  |  |
| NSCLC | 39 | 40 |
| SCLC | 35 | 28 |

Abbreviations: WBRT, whole-brain radiation therapy; SIB, simultaneous integrated boost; GPA, graded prognostic assessment; NSCLC, non-small cell lung cancer; SCLC, small cell lung cancer. †Two of the lung cancer patients in the study had an undefined pathological type, thus they were excluded from the stratified analysis.
