# Supplementary figures and images for: Adding simultaneous integrated boost to whole brain radiation therapy improved intracranial tumour control and minimize radiation-induced brain injury risk for the treatment of brain metastases
Source: BMC Cancer. 2023 Dec 16;23:1240. doi: 10.1186/s12885-023-11739-9 (PMC10724957; doi:10.1186/s12885-023-11739-9)

Supplementary Figure 1

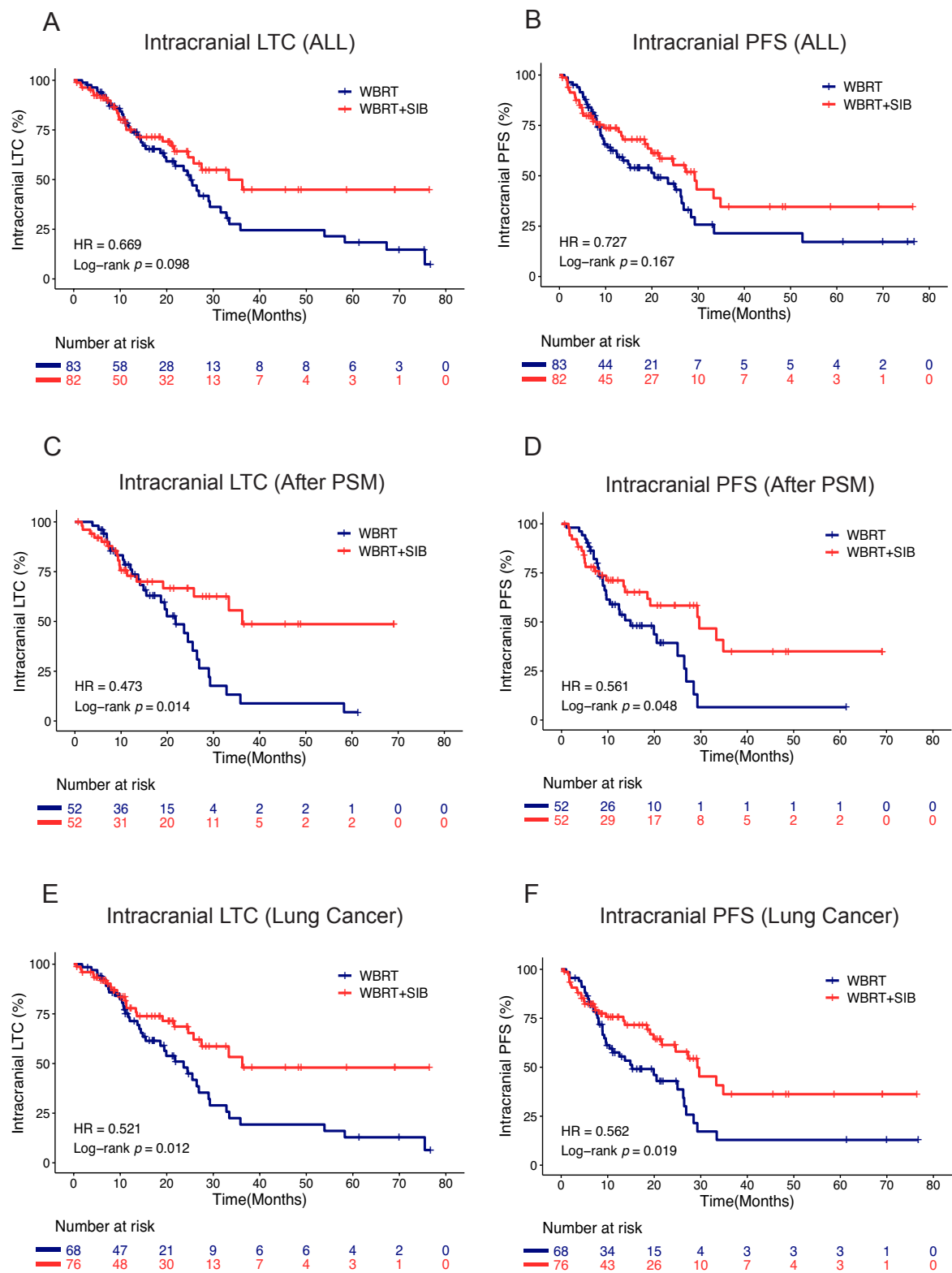

Supplement: Supplementary file 2 — Additional file 2. Supplementary Fig. 1 Comparison of intracranial local tumour control (LTC) (A) and progression-free survival (PFS) (B) between whole brain radiation therapy with a simultaneous integrated boost (WBRT + SIB) and whole brain radiation therapy (WBRT) in all patients using Kaplan-Meier method. Comparison of intracranial LTC (C) and PFS (D) between WBRT + SIB and WBRT after propensity score matching (PSM) using Kaplan-Meier method. Comparison of intracranial LTC (E) and PFS (F) between WBRT + SIB and WBRT in the lung cancer subgroup using Kaplan-Meier method. [file 12885_2023_11739_MOESM2_ESM.pdf]

Supplementary Figure 2

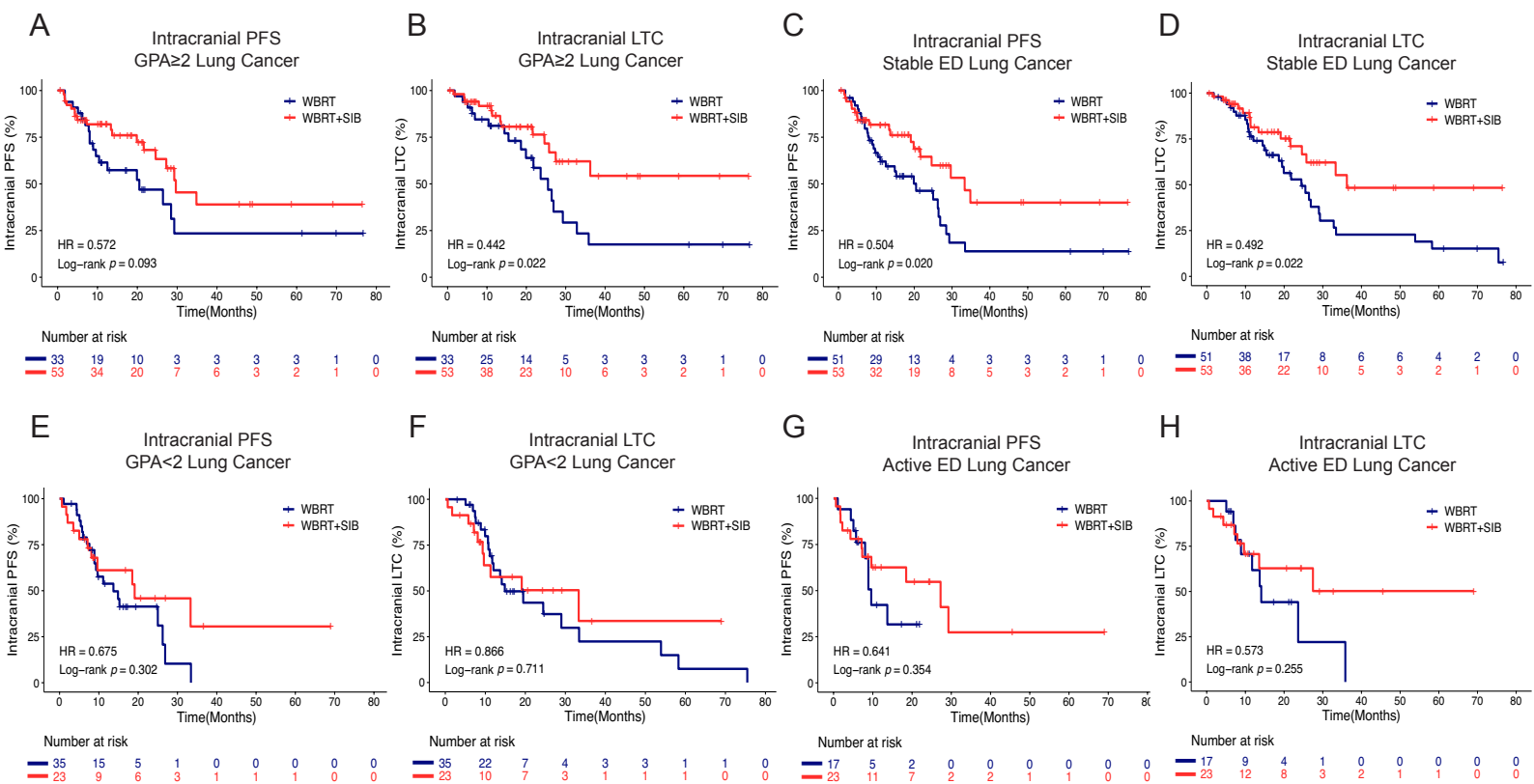

Supplement: Supplementary file 3 — Additional file 3. Supplementary Fig. 2 Comparison of intracranial progression-free survival (PFS) (A) and local tumour control (LTC) (B) for WBRT and WBRT + SIB in lung cancer patients with a graded prognostic assessment (GPA) score ≥ 2 using Kaplan-Meier method. Comparison of intracranial PFS (C) and LTC (D) for WBRT and WBRT + SIB in lung cancer patients with stable extracranial disease (ED) using Kaplan-Meier method. Comparison of intracranial PFS (E) and LTC (F) for WBRT and WBRT + SIB in lung cancer patients with a GPA < 2 using Kaplan-Meier method. Comparison of intracranial PFS (G) and LTC (H) for WBRT and WBRT + SIB in lung cancer patients with active ED using Kaplan-Meier method. [file 12885_2023_11739_MOESM3_ESM.pdf]

Supplementary Figure 3

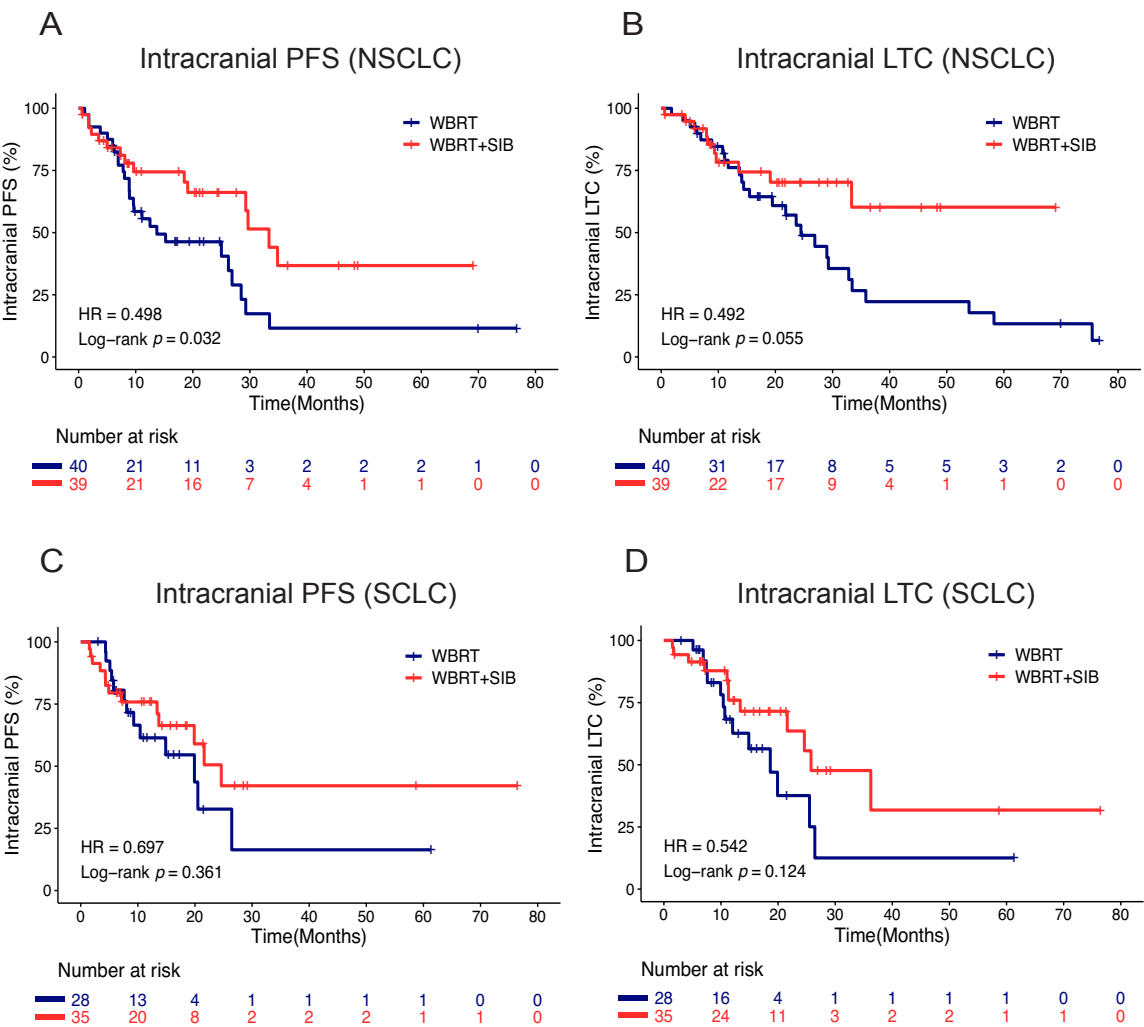

Supplement: Supplementary file 4 — Additional file 4. Supplementary Fig. 3 Comparison of intracranial progression-free survival (PFS) (A) and local tumour control (LTC) (B) for WBRT and WBRT + SIB in non-small cell lung cancer patients (NSCLC) using Kaplan-Meier method. Comparison of intracranial PFS (C) and LTC (D) for WBRT and WBRT + SIB in small cell lung cancer (SCLC) patients using Kaplan-Meier method. [file 12885_2023_11739_MOESM4_ESM.pdf]

Supplementary Figure 4

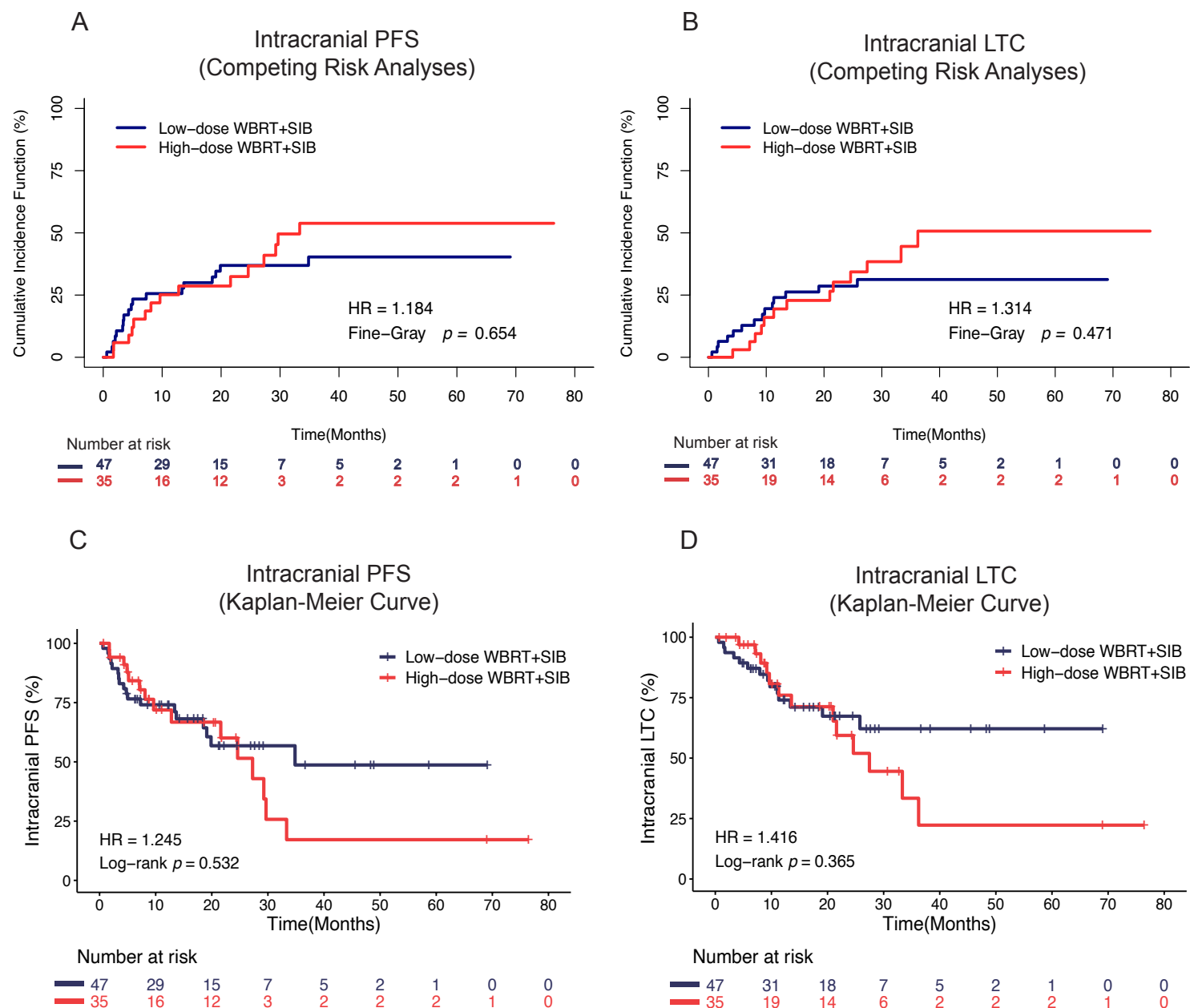

Supplement: Supplementary file 5 — Additional file 5. Supplementary Fig. 4 Comparison of intracranial progression-free survival (PFS) (A) and local tumour control (LTC) (B) for low-dose and high-dose WBRT + SIB group using competing risk analyses. Comparison of intracranial PFS (C) and LTC (D) for low-dose and high-dose WBRT + SIB group using Kaplan-Meier method. [file 12885_2023_11739_MOESM5_ESM.pdf]
